# Should IUI replace IVF as first-line treatment for unexplained infertility? A literature review

**DOI:** 10.1186/s12905-023-02717-1

**Published:** 2023-10-27

**Authors:** Jessica Ka-Yan Man, Anne Elizabeth Parker, Sophie Broughton, Hamza Ikhlaq, Mausumi Das

**Affiliations:** 1 Faculty of Medicine, Imperial College London (Hammersmith Campus), Du Cane Road, London, W12 0NN UK; 2 Medical School, College of Medical and Dental Sciences, University of Birmingham, Edgbaston, Birmingham, B15 2TT UK; 3 Queen Charlotte’s and Chelsea Hospital, Imperial College Healthcare NHS Trust, Du Cane Road, London, W12 0HS UK

**Keywords:** IUI, IVF, Infertility, Unexplained infertility, Ovarian stimulation, Pregnancy outcomes

## Abstract

**Background:**

Unexplained infertility accounts for 25% of infertility causes in the UK. Active intervention methods, such as intrauterine insemination (IUI) or in vitro fertilisation (IVF), are often sought. Despite the National Institute for Health and Care Excellence (NICE) recommending IVF for unexplained infertility, this recommendation has generated an ongoing debate, with few fertility clinics discontinuing the use of IUI as the first-line management of choice. In contrast to NICE, recent guidance released from the European Society for Human Reproduction and Embryology (ESHRE) in August 2023 supports the use of IUI as first-line. High-quality evidence behind such interventions is lacking, with current literature providing conflicting results.

**Aims:**

This review aims to provide a literature overview exploring whether IUI or IVF should be used as first-line treatment for couples with unexplained infertility, in the context of current guidelines.

**Methods:**

The primary outcome used to assess efficacy of both treatment methods is live birth (LB) rates. Secondary outcomes used are clinical pregnancy (CP) and ongoing pregnancy (OP) rates. A comprehensive literature search of 4 databases: Ovid MEDLINE, EMBASE, Maternity & Infant Care and the Cochrane Library were searched in January 2022. Upon removal of duplications, abstract screening, and full-text screening, a total of 34 papers were selected.

**Discussion/conclusion:**

This review highlights a large discrepancy in the literature when examining pregnancy outcomes of IUI and IVF treatments. Evidence shows IUI increases LB and CP rates 3-fold compared to expectant management. Literature comparing IUI to IVF is less certain. The review finds the literature implies IVF should be used for first-line management but the paucity of high-quality randomised controlled trials (RCTs), coupled with heterogeneity of the identified studies and a lack of research amongst women > 40 years warrants the need for further large RCTs. The decision to offer IUI with ovarian stimulation (IUI-OS) or IVF should be based upon patient prognostic factors. We suggest that IUI-OS could be offered as first-line treatment for unexplained infertility for women < 38 years, with good prognosis, and IVF could be offered first to those > 38 years. Patients should be appropriately counselled to enable informed decision making.

**Supplementary Information:**

The online version contains supplementary material available at 10.1186/s12905-023-02717-1].

## Background

Infertility affects an estimated 1 in 7 couples across the UK, of which 25% is due to unexplained causes [1]. Couples who are unable to conceive for at least one year and where standard investigations fail to identify any abnormalities in ovulation, tubal patency, and semen analysis are classed as experiencing unexplained infertility [2, 3]. Expectant management (EM) encourages natural conception through regular, unprotected sexual intercourse [4], but many couples with unexplained infertility prefer more active clinical interventions [5], such as intrauterine insemination (IUI) with ovarian stimulation (OS), and in-vitro fertilisation (IVF) [4]. IUI involves directly inserting sperm into the uterus at time of ovulation and can be performed in conjunction with OS to increase the number of available eggs at the site of fertilisation [4, 6]. Commonly used stimulation agents include clomiphene citrate (CC), letrozole and gonadotrophins [7]. Conversely, IVF engages fertilisation outside of the uterus and can involve various methodologies, such as intracytoplasmic sperm injection and transfer of differing numbers of embryos based on embryo quality [1, 2].

Globally, there is a discrepancy between the use of IUI or IVF as first-line treatment for unexplained infertility [5]. While IUI is widely considered less invasive and lower cost, its effectiveness and risks for multiple births are debated [7]. The UK National Institute for Health and Care Excellence (NICE) recommend offering IVF as first-line treatment to couples with unexplained infertility after 2 years of unsuccessful EM [1, 2, 4]. IUI is no longer recommended [4]. This 2013 guidance, acknowledged by NICE as based upon largely low-quality evidence [4], has been met with contention and only 4% of all UK gynaecologists have discontinued the use of IUI [5, 8]. Several systematic reviews have since concluded there is a lack of robust evidence in this area due to the imprecise and heterogeneous nature of the trials [7, 9]. Conversely, the newly released 2023 guideline from European Society of Human Reproduction and Embryology (ESHRE) recommends IUI with ovarian stimulation as first-line treatment for unexplained infertility [10, 11].

Therefore, in the context of currently conflicting guidelines, this review aims to explore current evidence on the practise of IUI versus IVF as first-line treatment for unexplained infertility via comparison of pregnancy outcomes. The primary outcome is live birth (LB) rates as the preferred outcome for evaluation of infertility treatment recommended by the ESHRE [12, 13]. Secondary outcomes are clinical pregnancy (CP) and ongoing pregnancy (OP) rates, to avoid exclusion of relevant studies given the large disparity in pregnancy outcomes reported within studies. This review limits literature to 2010 onwards to minimise analysis of outdated evidence prior to the well-documented increase in IVF success rates over the past decade [14, 15].

## Methods

**Fig. 1:**
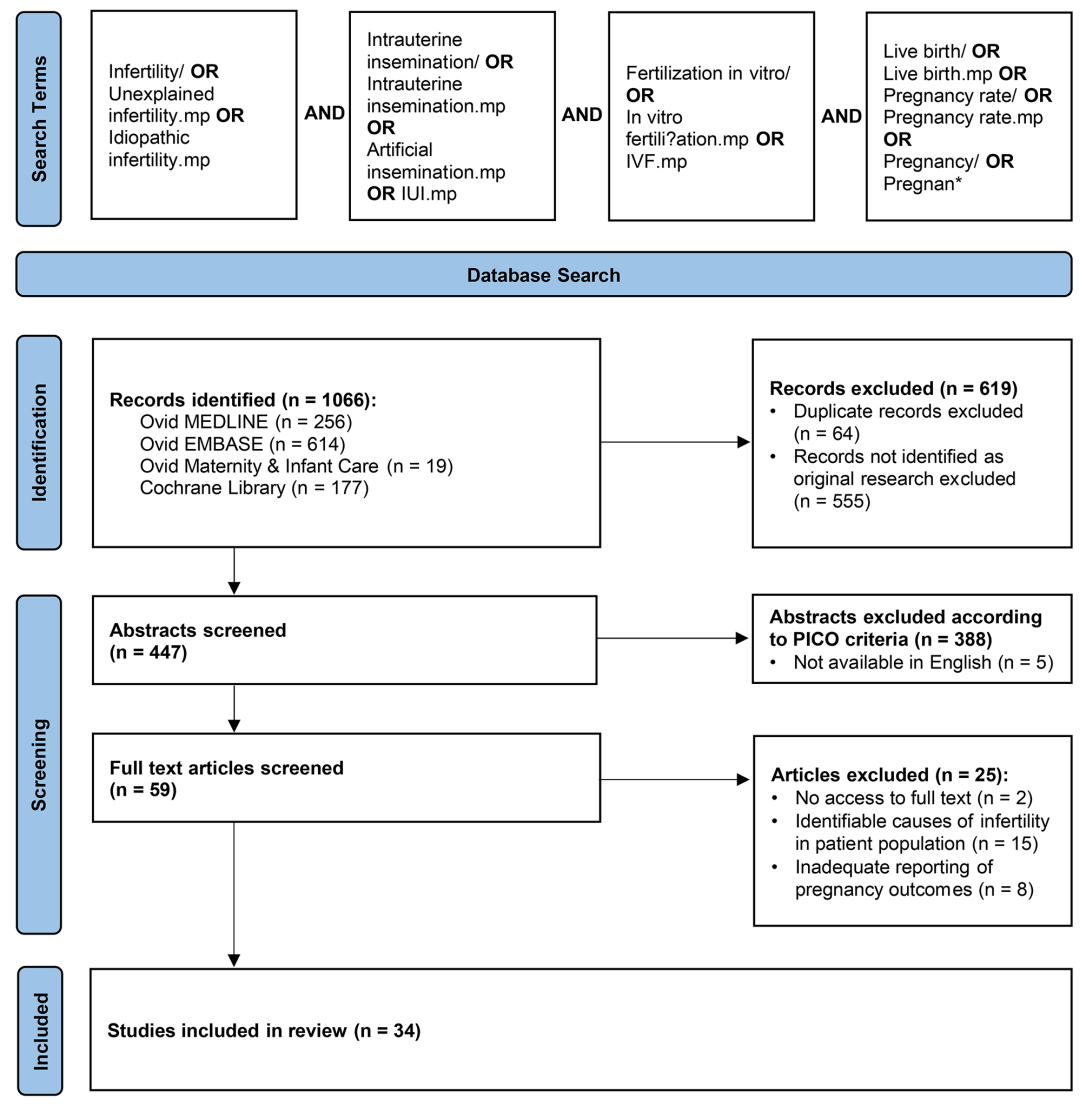
Modified PRISMA Flow Diagram of the literature search strategy used. Following database searching, a total of 1066 records were identified. Abstracts and full texts were screened, and a total of 34 articles were selected and included in this review. Inclusion criteria was any original research paper on humans published between 1 January 2010 to 6 January 2022 that was relevant to the research question. Any papers that met the following exclusion criteria were removed: not available in English (n = 5), no access to full-text version (n = 2), incomplete reporting of pregnancy outcomes (n = 8), included patients with identifiable causes of infertility (n = 15)

The PICO framework was used to develop this review question and the search strategy (Table 1). A literature search of Ovid MEDLINE, EMBASE, Maternity & Infant Care and Cochrane Library databases was conducted in January 2022 as outlined in Fig. 1. Following abstract and full-text screening, a total of 34 articles were selected for review.

**Table 1:** Review Question PICO Framework

Should IUI replace IVF as first-line treatment for couples with unexplained infertility?	
**Population**	Couples with unexplained infertility
**Intervention**	Intrauterine insemination (IUI) with or without ovarian stimulation (OS)
**Comparator**	In vitro fertilisation (IVF)
**Outcome(s)**	Primary outcome: Live birth (LB) rates Secondary outcome: Clinical pregnancy (CP) or ongoing pregnancy (OP) rates

## IUI and pregnancy outcomes

### IUI: live birth rates

6 papers evaluating the effects of IUI upon LB rates were identified, with 1 randomised controlled trial (RCT) and 5 retrospective observational studies [16–21]. The reliance on retrospective analysis diminishes the quality of evidence available and further RCTs are advised.

McLernon et al. reported couples with IUI-OS to be 3 times more likely to conceive than those with EM [14]. It was particularly more effective in couples with poorer prognosis. Prognosis was predicted via the validated Hunault model, which ‘estimates chances of natural conception leading to a LB within 12 months’ [16]. Although this retrospective study did not adjust for confounding of its differing baseline characteristics [16], the findings were corroborated by a RCT by Farquhar et al. (n = 201) [17]. This similarly found a 3-fold increase in cumulative LB rates in women with poor prognosis of < 30% chance of conception over 12 months [17]. Despite this trial’s strict exclusion criteria, however, several patients with > 30% prognosis and explained causes of infertility (e.g., polycystic ovary syndrome, PCOS) were also included, introducing selection bias and confounding within the results. However, the authors have justified the inclusion of PCOS population to ‘reflect diversity of women who have no clear explanation for their infertility’ [17]. Additionally, only 72% of couples in the EM group self-reported dates of sexual intercourse, suggesting potential poor compliance and response bias, although the statistical power and use of block randomisation in this RCT provides the highest quality evidence [17].

Although studies reporting cumulative LB rates have comparable results (26.5% versus 28.5%) [18, 19], inconsistencies exist in how rates were calculated with only one [19] adjusting for confounders, and no comparisons against EM could be made due to their lack of control groups. LB rates varied widely between 8.8% and 38% in the literature, but most were single-centre studies conducted in different countries and thus lack generalisability to clinical practice across the UK [18, 20, 21].

Multiple studies have explored the optimum number of IUI-OS cycles leading to a LB. The consensus is that an average 2 cycles of IUI-OS are required [18–21]. Multiparous women aged < 32 years on their first IUI-OS cycle had the best chances of LB with a probability of 21.4% [19, 20]. With more cycles, success rates progressively decline with adjusted odds of LB of 0.75 (95% confidence intervals, CI 0.62–0.93, p < 0.01) per additional cycle attempt [19]. Osmanlıoğlu Ş et al. report up to 3 cycles are effective, yet only 27% of their cohort had a third cycle with more opting for IVF treatment after a failed second cycle [18]. Many were limited in sample size, due to high numbers of unexplained dropout rates [18, 19, 21].

The NICE guidelines state that OS agents should not be offered to women with unexplained infertility [4], although IUI-OS has been shown to increase LB [16, 17, 20]. Huang et al. and Osmanlıoğlu Ş et al. achieved similar LB rates between stimulation methods [18], however Christie et al. found IUI-OS with human menopausal gonadotrophin (HMG) led to nearly 3 times higher LB than IUI-OS with CC [21]. This study had similar proportions of IUI-HMG cycles and IUI-CC cycles (40.9% versus 45.3%) but was limited by large 95% CI (0.78–8.80), small sample size (n = 72 versus 65 cycles) and selection bias due to its setting in Jamaica where no financial support for fertility treatment is available and CC is less costly than HMG [21]. While IUI-OS increases LB, the choice of OS is usually down to the clinician’s and patient’s discretion [17]. These conflicting results warrant further research into the efficacy of each stimulation method with higher-quality RCTs.

Unlike the NICE 2013 guidelines [1, 4], the recent ESHRE 2023 guidelines support the use of ovarian stimulation combined with IUI [10, 11]. Although the authors found that there was no significant difference in LB rates between unstimulated IUI and EM (23% vs. 16%), IUI-OS was recommended as first-line treatment for unexplained infertility [10, 11]. This strong recommendation was based on a 2020 systematic review and meta-analysis, including 4 RCTs, by Ayeleke et al. [7], who reported higher LB rates with IUI-OS compared with IUI in a natural cycle (OR 2.07, 95% CI 1.22–3.50). These conclusions were made with uncertainty due to the nature of the very low to moderate quality trials included [7]. Compared to EM, success rates in LBs with IUI-OS were higher in couples with poor prognosis (OR 4.48, 95% CI 2.00-10.01) than in those with moderate prognosis (OR 0.82, 95% CI 0.45–1.49), so it was advised that the decision to start active treatment should be based on patient prognosis and preferences [10, 11]. Poor prognosis was defined by a prediction score of natural conception of less than 30% and moderate prognosis as a score between 30% and 40% [7], yet ESHRE recognised that the Hunault prediction model has only been validated for the Canadian and Dutch population, and more importantly, is limited by use at the point of diagnosis only [10, 11].

### IUI: clinical pregnancy and ongoing pregnancy rates

The literature search identified 12 papers addressing IUI with CP and/or OP rates, including: 3 RCTs, 7 cohort studies, 1 observational study and 1 prospective analysis [17–28]. The effects of IUI upon both CP and OP rates are well documented. However, discrepancies exist when defining pregnancy rates between papers. There remains little guidance on a standardised approach to reporting pregnancy outcomes with an appeal for emphasis on singleton live births [29]. However, studies reporting CP and OP are not necessarily of lower validity than those with LB as they allow for interim analyses months earlier, which can allow for couples to transfer to alternative treatments if one is shown to be much more efficacious than the other [30].

The literature suggests that IUI-OS is an effective treatment for women with unexplained infertility as compared to EM [17, 18, 22]. The previously mentioned RCT by Farquhar et al. also reported a 3-fold increase in CP rates associated with IUI-OS compared to EM [17]. Similar results have been observed across retrospective and prospective cohort studies of lower power and smaller sample sizes [18, 21–23]. Although this evidence supports increased CP rates with IUI-OS, most of the literature only studied women below the age of 40. Furthermore, Farquhar et al. aimed to study women aged up to 42 years, however, no women over 40 were included [17]. Evidence on the use of IUI-OS in women aged above 40 years is limited and lacks specific NICE guidance, despite maternal age being an important prognostic factor for a successful pregnancy [21, 22]. Of the evidence that is available within this age group, Wiser et al. reported low CP rates with IUI-OS and concluded it is not the most effective treatment for women over 40 years old [24], although this was a retrospective study and further prospective trials are recommended.

A large retrospective cohort study (n = 851) concluded IUI-OS to be well-tolerated, with 37.4% CP rate over 3 cycles and patient preference for IUI-OS over the invasiveness of IVF [19]. Therefore, the clinical applicability of IUI-OS as an intervention to increase CP rates is promising. However, this study lacked a control EM group which limited the external validity of the findings. Widely, literature reported higher pregnancy rates with IUI-OS as compared to EM [17, 18, 21, 22], although, one RCT (n = 253) by Custers et al. found no difference between cumulative OP rates [25], yet this trial only compared IUI-OS to EM over 6 months. Furthermore, the number of IUI-OS cycles per couple during this timeframe was not stated, suggesting potential reporting bias. Numerous cycles of IUI-OS over a longer timeframe are widely considered necessary to achieve pregnancy [26].

Several studies did not examine differences between forms of OS; further trials would benefit from exploring forms of OS on the effects of increasing pregnancy rates with IUI [22, 23]. It is evident from the literature identified that IUI provides a promising outcome for increasing pregnancy rates amongst people struggling with unexplained infertility [17, 18, 21, 22]. However, there is a reliance on cohort studies and more high-quality RCTs are needed to further assess clinical efficacy and practical implementation of IUI.

## IUI vs. IVF and pregnancy outcomes

### IUI vs. IVF: live birth rates

15 studies compared IUI and IVF with LB rates, with 8 RCTs, 2 cohort studies, 2 longitudinal studies, 1 cross-sectional study and 2 retrospective analyses [31–45].

5 RCTs and 1 cohort study concluded that IUI-OS should be offered as first-line treatment over IVF [40–45]. Rather than finding higher LB with IUI-OS, studies found that LB rates did not differ [40–44]; as such, IUI-OS was favoured due to cost-effectiveness and high patient tolerance. A non-inferiority RCT conducted by Bensdorp et al. [44] further subdivided IVF single embryo transfer (IVF-SET) and modified natural cycle (IVF-MNC) as compared to IUI-OS, with no difference upon LB identified. This trial had high external validity by allowing couples from 17 centres to alter their treatment options beyond a time horizon of 1 year [44]. However, like several other studies [40, 41, 43, 45], the trial suffered from poor representation of unexplained infertility populations and an insufficient follow-up period to allow for cycles to be fully completed and long-term efficacy to be determined [44]. Resultantly, final conclusions made in support of IUI-OS were based on economic factors [17, 40, 41,43], safety considerations [40, 41, 43, 44], and without incorporation of EM [40, 42, 43], rather than reliable efficacy findings.

The Fast Track and Standard Treatment (FASTT) trial, a 2010 RCT, compared participants initially undergoing 3 cycles of IUI-OS with CC before embarking on treatment pathways of either: 3 cycles IUI-OS with FSH before 6 cycles of IVF (n = 247) or solely 6 cycles of IVF (n = 256) [31]. Although this trial did not directly compare LB rates, its primary outcome measured time to pregnancy leading to a LB. FASTT strongly favours the early implementation of IVF (HR 1.25; 95% CI 1.00-1.56), strengthened by its large sample size and robust single-centre standardisation protocols [31]. However, since this trial concluded in 2006, LB rate per embryo transferred has steadily risen from approximately 18–32% for IVF patients under 35 [15], an age category representing 64.2% of participants enrolled on FASTT [31]. Given that the average age of an IVF patient is now 35.7 years [15], it is likely that these findings are no longer accurately applicable to current clinical practice. This is reinforced by the aforementioned 2015 RCT conducted by Bensdorp et al. [44], which instead concluded no difference in time to pregnancy leading to LB for IVF (IVF-SET p = 0.38, IVF-MNC p = 0.59) when compared to IUI-OS. Furthermore, retrospective studies have since been conducted on the same FASTT participants, showing that 20.3% of patients later received an alternative infertility diagnosis [32, 33], removing them from the true unexplained infertility population (along with 3.6% initially identified to have infertility diagnoses [31]). This limits the generalisability of these findings.

Retrospective studies and RCTs with smaller sample sizes have attempted to corroborate these initial FASTT recommendations for the early, direct implementation of IVF rather than IUI-OS (CC, FSH), whether in a combined or stand-alone approach [34–36]. Unfortunately, these studies are of limited validity due to methodological inconsistencies, high dropout rates and differing baseline characteristics [34–36]. However, an RCT conducted by Elzeiny et al. [37], founded upon stringently inclusive criteria for both patient recruitment and statistical analyses, successfully mirrored FASTT recommendations, reporting LB rates of 40% IVF vs. 6% IUI-OS (RR 6.6, 95%CI 1.6–13.0, p = 0.01). Similarly high-quality studies are required to clarify efficacy and optimum number of cycles.

The ESHRE guidelines [10, 11] also reported that IVF is associated with higher LB rates than 3 months of EM (OR 22.0), and therefore recommends IVF over EM despite the very large 95% CI (2.56-189.38) and small sample size (n = 51, 1 RCT). LB rates were also higher following IVF compared to unstimulated IUI (OR 2.47, 95% CI 1.19–5.12) based on 156 women from 2 RCTs [11]. Furthermore, LB rates were found to be significantly higher after IVF treatment compared to IUI-OS (RR 1.54, 95% CI 1.04–2.28) [11]. Despite this evidence from a 2022 systematic review and meta-analysis by Nandi et al. [46], which included 7 RCTs and 1391 women, the ESHRE states that IVF is probably not recommended over IUI-OS [10, 11]. IUI-OS was regarded as non-inferior to IVF given the need for consideration of additional costs and risks associated with IVF treatment. This was supported by evidence from a sensitivity analysis that reported no significant difference in LB rates in women without previous treatment and < 38 years (RR 1.01, 95% CI 0.88–1.15) [10, 11]. However, in older women ≥ 38 years, IVF treatment was superior to IUI-OS (RR 2.15, 95% CI 1.16-4.00), so it was recommended to make patient-individualised decisions regarding considering IVF treatment [10, 11]. Overall, there does remain a paucity in the number of studies for women > 38 years of age. The most recent 2023 systematic review and meta-analysis in this older age group failed to make conclusions on the relative effectiveness of IVF and IUI due to insufficient evidence. Despite this, their meta-regression results seem to support the initial use of IVF over IUI, with IUI for women > 43 years of age resulting in a pregnancy rate < 5% compared to LB rates of 20% at 38 years, to 5% at 43 years, and 0% ≥ 44 years with IVF treatment [47].

### IUI vs. IVF: clinical pregnancy and ongoing pregnancy rates

13 studies reported CP and/or OP rates with IUI-OS and IVF, with 7 RCTs, 4 retrospective cohorts and 2 prospective cohorts [31, 34–38,40, 41–44, 48, 49]. The differing outcomes create a mixed picture of evidence; all studies reporting OP concluded no difference between IUI-OS and IVF [31, 40–42, 44, 48], while studies reporting CP had a higher number in favour of IVF [34–38].

All RCTs favouring IUI-OS found no difference between IVF and IUI-OS but argued IUI-OS is less invasive and less expensive with higher patient tolerance [40, 41, 44]. This is comparable to evidence with LB. 3 RCTs found higher pregnancy rates following IVF, with CP rates ranging from 40.0 to 49.0% with IVF versus 12.0–21.6% with IUI [31, 34, 37]. However, these trials were of varying quality with a wide range of sample sizing (n = 43 to n = 503)[31, 34, 37]. The Elzeiny et al. RCT found higher CP rates following IVF (RR 3.3, 95% CI 1.02–6.3) but their small sample size (n = 43) produced wide CI and one pregnancy difference between IVF and IUI-OS arms would have removed all statistical significance [37]. The authors acknowledge this and suggest larger sample sizing in future research is necessary.

Follow-up length and number of cycles differed between studies, which may account in part for the variation in results. Tjon-Kon-Fat et al. published a high-quality, multi-centre RCT with no difference in OP nor LB rates between treatments but had limited their trial to a 12-month analysis post first treatment [40]. This may not allow for sufficient time to achieve pregnancy and therefore produces a premature analysis with limited generalisability to clinical practice. Comparison of studies is further made difficult as some provide several cycles of IUI-OS [31, 35–37, 40, 42–44, 48] and IVF before analysis, but some only compare per cycle [34, 38, 41, 49].

The cohort studies were limited in quality by their design and retrospective nature [35, 36, 38, 43, 48, 49]. One prospective cohort reported patients with unexplained infertility but did not stratify their results from all causes of infertility, as such no findings applicable to the research question could be appraised [49]. Some of the cohort studies included differing baseline patient characteristics, which introduced selection bias and limited the validity of their findings [35, 36]. Merviel et al. reported higher CP per cycle with IVF (OR 4.20, 95% CI 3.72–4.68) but the baseline age, male BMI and duration of infertility differed between IVF and IUI-OS arms [38], thus reducing the reliability of the data.

Similarly to LB rates, the ESHRE guideline also finds that 1 cycle of IVF is associated with higher CP rates compared to 3–6 months of EM (OR 3.24, 95% CI 1.07–9.80) based on 2 RCTs, but due to its very low quality, the evidence was deemed inconclusive [10, 11, 50]. Overall reporting of CP and OP rates across studies show discrepancies in findings, with some reporting higher pregnancy rates with IVF and others reporting no difference between IVF and IUI. Evidence comparing CP and OP outcomes were not addressed in the ESHRE guidelines.

## Risk of multiple pregnancy

Concerns over IUI-OS as first-line treatment is mainly attributed to its risk of MP, which is associated with considerable maternal and neonatal morbidity, in addition to substantial financial burden [41].

Evidence of varying quality demonstrated comparably low MP rates following IUI-OS and IVF, in which most were twin pregnancies [19, 31, 38, 41, 44, 45, 51]. This could be explained by the implementation of strict cancellation criteria adopted for IUI-OS to prevent higher-order MP. IUI-OS cycles were withheld after a specific number of follicles had reached a particular diameter. This protocol varied between studies, so any discrepancies in MP rates may be due to the degree of stringency and threshold differences for defining a mature follicle diameter, of which ≥ 14 mm was most commonly used [52]. IUI-OS is usually limited to 1–2 follicles to prevent increased MP rates [25], yet Verhaeghe et al. [52] reported no significant differences in MP rates with 1–2 follicles versus 3–4 mature follicles on trigger day (18.2% versus 10%, p = 0.99). It was also the first to report MP risk after IVF to IUI-OS in women. This, however, included women with diminished or poor ovarian reserve, which may skew the observed low twin pregnancy rates (14.3%) [52]; so more conclusive prospective RCTs are needed.

The literature identified suggests acceptably low MP rates can be achieved with IUI-OS, with the implementation of strict cancellation criteria. However, future research and clinical practice should look to determine more standardised criteria across countries worldwide. No significant differences in multiple pregnancy rates were reported between IVF and IUI-OS treatment in systematic reviews by Pandian et al. (RR 1.03, 95% CI 0.04–27.29) [50], and Nandi et al. (RR 0.83, 95% CI 0.50–1.38) [46], but the ESHRE guidelines still does recognise the need for care to avoid multiple pregnancies by using a low-dose gonadotrophin treatment regimen [10, 11].

## Limitations

This review has highlighted several limitations within the literature identified by our search. Firstly, there was no standardisation in the definitions of unexplained infertility and pregnancy outcomes between samples. This is particularly evident for pregnancy rates where definitions vary widely. Further heterogeneity was also observed amongst the diagnostic and inclusion criteria of the selected studies with international comparisons being difficult to make. The literature identified is skewed towards cohort studies, mainly of a retrospective design, emphasising the lack of high-powered RCTs. Studies associated with fertility treatments often have high dropout rates, of which emotional burnout and time commitments were frequently cited as reasons for discontinuation [20, 39]. Given the variable time to successful conception [31, 39, 42], a holistic approach is paramount to ensuring a supportive environment throughout a couple’s fertility journey.

This review itself has limitations with regards to subsets of assisted reproductive techniques, and EM with OS was not addressed. This work was also unable to evaluate IUI without OS as literature regarding its efficacy upon pregnancy outcomes was minimal. Additionally, it may have been useful to standardise the following methodologies: agents and doses used in OS, timing of insemination within the menstrual cycle, acceptable fertility prognosis as well as algorithmic methods and tools used to calculate this. A variety of maternal and neonatal outcomes were also not addressed, such as incidence of ovarian hyperstimulation syndrome, mode of delivery and preterm birth rates. Evaluation of cost-effectiveness was outside the scope of this review and discrepancies between national healthcare models may yield varying analysis, although widely agreed that upfront costs of IVF are higher [40, 41], some studies have identified IVF is more cost-effective than IUI-OS on a cost per live birth basis [37, 40].

## Conclusions

Upon a review of the existing literature, IUI-OS largely demonstrates improved LB, CP and OP outcomes when compared to EM. Studies comparing IUI-OS and IVF were altogether inconclusive; with some studies reporting increased LB, CP and OP outcomes with IVF and others finding no difference between the interventions. Yet, seminal research to support IVF is over 15 years old and more recent research concludes that IUI-OS should be favoured, albeit due to higher patient tolerance and lower cost rather than differences in efficacy. As supported by systematic reviews and meta-analyses, further RCTs are warranted [9]. Future research should stratify results for patients with unexplained infertility via their baseline ovarian reserve, age, and prognosis with stringent exclusion criteria to limit heterogeneity. Further high-quality research may better reflect the evolving trends in IVF success rates upon pregnancy outcomes to demonstrate advancements in efficacy, safety and cost-effectiveness [15].

Future work may support emerging evidence from ESHRE regarding the use of IUI-OS as first-line treatment. This review identified a paucity of information regarding the use of active treatments amongst women above the age of 40 which warrants further study in the contexts of unexplained infertility. Additionally, this review identified the risk of multiple pregnancy and the importance of implementing risk counselling as important considerations for the clinical applicability of these interventions. However, in the interim of awaiting more conclusive evidence, clinics should continue to offer both interventions, especially as we await further review of NICE guidance in light of the emerging literature presented by ESHRE and presented in this review. The decision to offer IUI-OS or IVF should be based upon factors such as the woman’s age, duration of infertility, existing pathologies, previous fertility treatments and previous pregnancies. We suggest that IUI-OS could be offered to women with unexplained infertility who are less than 38 years with good prognosis, and IVF could be offered first to those greater than 38 years of age. Patients should be appropriately counselled to enable them to make an informed decision.

### Electronic supplementary material

Below is the link to the electronic supplementary material.

Supplementary Material 1

## Data Availability

All data generated or analysed in this review are available from Ovid Technologies MEDLINE, Embase and Maternity & Infant Care databases, and Cochrane Library repository, and are included in this published article and its supplementary information file.

## Abbreviations

IUI: intrauterine insemination
IVF: in-vitro fertilisation
LB: live birth
CP: clinical pregnancy
OP: ongoing pregnancy
EM: expectant management
OS: ovarian stimulation
CC: clomiphene citrate
NICE: National Institute for Clinical Excellence
ESHRE: European Society for Human Reproduction and Embryology
RCT: randomised controlled trial
PCOS: polycystic ovary syndrome
HMG: human menopausal gonadotrophin
SET: single embryo transfer
MNC: modified natural cycle
FASTT: Fast Track and Standard Treatment trial

## References

[1] *National Institute for Health and Care Excellence (NICE), Fertility problems: assessment and treatment: Clinical Guideline [CG156]* https://www.nice.org.uk/guidance/cg156] [Accessed Jan 14, 2022]. 31804780

[2] Quaas A, Dokras A (2008). Diagnosis and treatment of unexplained infertility. Reviews in Obstetrics and Gynecology.

[3] World Health Organization. *Infertility* https://www.who.int/news-room/fact-sheets/detail/infertility] [Accessed Jan 24, 2022].

[4] *National Institute for Health and Care Excellence (NICE), Addendum to Clinical Guideline 156, Fertility problems: assessment and treatment: Clinical Guideline Addendum 156.1* https://www.nice.org.uk/guidance/cg156/evidence/addendum-pdf-2606775661] [Accessed Jan 14, 2022]. 31804780

[5] Nandi A, Gudi A, Shah A, Homburg R (2015). An online survey of specialists’ opinion on first line management options for unexplained subfertility. Hum Fertility.

[6] Cohlen B, Bijkerk A, Van der Poel S, Ombelet W (2018). IUI: review and systematic assessment of the evidence that supports global recommendations. Hum Reprod Update.

[7] Ayeleke RO, Asseler JD, Cohlen BJ, Veltman-Verhulst SM (2020). Intra‐uterine insemination for unexplained subfertility. Cochrane Database of Systematic Reviews.

[8] Tjon-Kon-Fat RI, Bensdorp AJ, Scholten I, Repping S, van Wely M, Mol BWJ (2016). IUI and IVF for unexplained subfertility: where did we go wrong?. Hum Reprod (Oxford England).

[9] Wang R, Danhof NA, Tjon-Kon‐Fat RI, Eijkemans MJ, Bossuyt PM, Mochtar MH (2019). Interventions for unexplained infertility: a systematic review and network meta‐analysis. Cochrane Database Syst Rev.

[10] Romualdi D, Ata B, Bhattacharya S, Bosch E, Costello M, Gersak K, et al. Evidence-based guideline: unexplained infertility. Hum Reprod (Oxford England). 2023;dead150. 10.1093/humrep/dead150]. 10.1093/humrep/dead150 PMC10546081 37599566

[11] Guideline Group on Unexplained Infertility, Romualdi D, Ata B, Bhattacharya S, Bosch E, Costello M et al. *Evidence-based guideline: Unexplained Infertility* https://www.eshre.eu/-/media/sitecore-files/Guidelines/UI/UI-guideline_-Final.pdf] [Accessed Sep 10, 2023]. 10.1093/humrep/dead150 PMC10546081 37599566

[12] Land JA, Evers JLH (2003). Risks and Complications in assisted reproduction techniques: report of an ESHRE consensus meeting. Hum Reprod (Oxford England).

[13] Barnhart KT (2014). Live birth is the correct outcome for clinical trials evaluating therapy for the infertile couple. Fertil Steril.

[14] Ombelet W, van Eekelen R, McNally A, Ledger W, Doody K, Farquhar C (2020). Should couples with unexplained infertility have three to six cycles of intrauterine insemination with ovarian stimulation or in vitro fertilization as first-line treatment?. Fertil Steril.

[15] Human F, Embryology Authority. &. *Fertility treatment 2019: trends and figures | HFEA* https://www.hfea.gov.uk/about-us/publications/research-and-data/fertility-treatment-2019-trends-and-figures/] [Accessed Jan 14, 2022].

[16] McLernon DJ, Lee AJ, Maheshwari A, van Eekelen R, van Geloven N, Putter H (2019). Predicting the chances of having a baby with or without treatment at different time points in couples with unexplained subfertility. Hum Reprod (Oxford England).

[17] Farquhar CM, Liu E, Armstrong S, Arroll N, Lensen S, Brown J (2018). Intrauterine insemination with ovarian stimulation versus expectant management for unexplained infertility (TUI): a pragmatic, open-label, randomised, controlled, two-centre trial. Lancet (London England).

[18] Osmanlıoğlu Ş, Şükür YE, Tokgöz VY, Özmen B, Sönmezer M, Berker B (2022). Intrauterine insemination with ovarian stimulation is a successful step prior to assisted reproductive technology for couples with unexplained infertility. J Obstet Gynaecology: J Inst Obstet Gynecol.

[19] Geisler ME, Ledwidge M, Bermingham M, McAuliffe M, McMenamin MB, Waterstone JJ (2017). Intrauterine insemination-No more Mr. N.I.C.E. guy?. Eur J Obstet Gynecol Reprod Biol.

[20] Ohannessian A, Loundou A, Gnisci A, Paulmyer-Lacroix O, Perrin J, Courbiere B (2017). Unexplained infertility: live-birth’s prognostic factors to determine the ART management. Minerva Ginecol.

[21] Christie LR, Harriott JA, Dacosta VE, Wynter SH, Everett DM, Foster RA (2011). Intrauterine insemination in Jamaica as a low-cost subfertility treatment in a low-resource region. Int J Gynaecol Obstet.

[22] Ganguly I, Singh A, Bhandari S, Agrawal P, Gupta N. Pregnancy Predictors after Intrauterine Insemination in Cases of Unexplained Infertility: A Prospective Study. *International Journal of Reproductive Medicine* 2016; 2016 5817823. 10.1155/2016/5817823]. 10.1155/2016/5817823 PMC5050366 27738654

[23] van Eekelen R, van Geloven N, van Wely M, McLernon DJ, Mol F, Custers IM (2019). Is IUI with ovarian stimulation effective in couples with unexplained subfertility?. Hum Reprod (Oxford England).

[24] Wiser A, Shalom-Paz E, Reinblatt SL, Son W, Das M, Tulandi T (2012). Ovarian stimulation and intrauterine insemination in women aged 40 years or more. Reprod Biomed Online.

[25] Custers IM, van Rumste MME, van der Steeg JW, van Wely M, Hompes PGA, Bossuyt P (2012). Long-term outcome in couples with unexplained subfertility and an intermediate prognosis initially randomized between expectant management and immediate treatment. Hum Reprod (Oxford England).

[26] Babigumira JB, Sharara FI, Garrison Louis P (2018). Projecting the potential impact of the Cap-Score™ on clinical pregnancy, live births, and medical costs in couples with unexplained infertility | journal of assisted Reproduction and Genetics. J Assist Reprod Genet.

[27] Dinelli L, Courbière B, Achard V, Jouve E, Deveze C, Gnisci A (2014). Prognosis factors of pregnancy after intrauterine insemination with the husband’s sperm: conclusions of an analysis of 2,019 cycles. Fertil Steril.

[28] Wang ET, Diamond MP, Alvero R, Casson P, Christman GM, Coutifaris C (2020). Androgenicity and fertility treatment in women with unexplained infertility. Fertil Steril.

[29] Braakhekke M, Kamphuis EI, van Rumste MM, Mol F, van der Veen F, Mol BW (2014). How are neonatal and maternal outcomes reported in randomised controlled trials (RCTs) in reproductive medicine?. Hum Reprod (Oxford England).

[30] Braakhekke M, Kamphuis EI, Dancet EA, Mol F, van der Veen F, Mol BW (2014). Ongoing pregnancy qualifies best as the primary outcome measure of choice in trials in reproductive medicine: an opinion paper. Fertil Steril.

[31] Reindollar RH, Regan MM, Neumann PJ, Levine B, Thornton KL, Alper MM (2010). A randomized clinical trial to evaluate optimal treatment for unexplained infertility: the fast track and standard treatment (FASTT) trial. Fertil Steril.

[32] Vaughan DA, Goldman MB, Koniares KG, Nesbit CB, Toth TL, Fung JL (2022). Long-term reproductive outcomes in patients with unexplained infertility: follow-up of the fast track and Standard Treatment Trial participants. Fertil Steril.

[33] Vaughan DA, Goldman MB, Fung JL, Koniares KG, Nesbit CB, Toth TL (2020). Long Term follow up of Reproductive outcomes in FASTT participants. Fertil Steril.

[34] Goldman MB, Thornton KL, Ryley D, Alper MM, Fung JL, Hornstein MD (2014). A randomized clinical trial to determine optimal infertility treatment in older couples: the forty and over treatment trial (FORT-T). Fertil Steril.

[35] Mendieta MAd, Aranda AS, López JFM, Sánchez AMR, Mondragón EC, Monterrosas LÁD (2022). Comparative analysis of the pregnancy rate via in vitro fertilization vs. previous artificial insemination in patients with unexplained infertility. JBRA Assist Reprod.

[36] Pacu I, Ionescu C, Dimitriu M, Banacu M, Tarcomnicu IM, Călin D et al. Intrauterine insemination in idiopathic infertility. *Archives of the Balkan Medical Union* 2016; 51: 334–339. https://www.researchgate.net/publication/313047153_Intrauterine_insemination_in_idiopathic_infertility]. [Accessed 14 January 2022].

[37] Elzeiny H, Garrett C, Toledo M, Stern K, McBain J, Baker HWG (2014). A randomised controlled trial of intra-uterine insemination versus in vitro fertilisation in patients with idiopathic or mild male infertility. Aust N Z J Obstet Gynaecol.

[38] Merviel P, Labarre M, James P, Bouée S, Chabaud J, Roche S (2022). Should intrauterine inseminations still be proposed in cases of unexplained infertility? Retrospective study and literature review. Arch Gynecol Obstet.

[39] Wu Y, Liu H, Liu J (2021). The Livebirth Rate Per in Vitro Fertilization cycle is higher than the Cumulative Live Birth Rates of Intrauterine Insemination for Patients of Poseidon Group 3 with unexplained infertility. Front Endocrinol.

[40] Tjon-Kon-Fat RI, Bensdorp AJ, Bossuyt PMM, Koks C, Oosterhuis GJE, Hoek A (2015). Is IVF-served two different ways-more cost-effective than IUI with controlled ovarian hyperstimulation?. Hum Reprod (Oxford England).

[41] van Rumste MME, Custers IM, van Wely M, Koks CA, van Weering HGI, Beckers NGM (2014). IVF with planned single-embryo transfer versus IUI with ovarian stimulation in couples with unexplained subfertility: an economic analysis. Reprod Biomed Online.

[42] Tjon-Kon-Fat RI, Tajik P, Zafarmand MH, Bensdorp AJ, Bossuyt PMM, Oosterhuis GJE (2017). IVF or IUI as first-line treatment in unexplained subfertility: the conundrum of treatment selection markers. Hum Reprod (Oxford England).

[43] Nandi A, Bhide P, Hooper R, Gudi A, Shah A, Khan K (2017). Intrauterine insemination with gonadotropin stimulation or in vitro fertilization for the treatment of unexplained subfertility: a randomized controlled trial. Fertil Steril.

[44] Bensdorp AJ, Tjon-Kon-Fat RI, Bossuyt PMM, Koks CM, Oosterhuis GJE, Hoek A, et al. Prevention of multiple pregnancies in couples with unexplained or mild male subfertility: randomised controlled trial of in vitro fertilisation with single embryo transfer or in vitro fertilisation in modified natural cycle compared with intrauterine insemination with controlled ovarian hyperstimulation. BMJ (Clinical Research ed). 2015;350g7771. 10.1136/bmj.g7771]. 10.1136/bmj.g7771 PMC4288434 25576320

[45] Malchau SS, Henningsen AA, Loft A, Rasmussen S, Forman J, Nyboe Andersen A (2017). The long-term prognosis for live birth in couples initiating fertility treatments. Hum Reprod (Oxford England).

[46] Nandi A, Raja G, White D, Tarek E (2022). Intrauterine insemination + controlled ovarian hyperstimulation versus in vitro fertilisation in unexplained infertility: a systematic review and meta-analysis. Arch Gynecol Obstet.

[47] Rake J, Mochtar M, Van Putten E, Van Wely M, P-789. Unexplained subfertility in women aged 38 years and above: could intrauterine insemination be an alternative for in vitro fertilization? A systematic review and meta-analysis. Hum Reprod. 2023;38(Supplement 1). 10.1093/humrep/dead093.144]. dead093.144.

[48] Brandes M, Hamilton CJCM, van der Steen JOM, de Bruin JP, Bots RSGM, Nelen WLDM (2011). Unexplained infertility: overall ongoing pregnancy rate and mode of conception. Hum Reprod (Oxford England).

[49] Smith JF, Eisenberg ML, Millstein SG, Nachtigall RD, Sadetsky N, Cedars MI et al. Fertility treatments and outcomes among couples seeking fertility care: data from a prospective fertility cohort in the United States. *Fertility and Sterility* 2011; 95 (1): 79–84. https://doi.org/1016/j.fertnstert.2010.06.043. 10.1016/j.fertnstert.2010.06.043 PMC2966858 20659733

[50] Pandian Z, Gibreel A, Bhattacharya S. In vitro fertilisation for unexplained subfertility. Cochrane Database of Systematic Reviews. 2015;11CD003357. 10.1002/14651858.CD003357.pub4]. 10.1002/14651858.CD003357.pub4 PMC7154339 26583517

[51] Custers IM, König TE, Broekmans FJ, Hompes PGA, Kaaijk E, Oosterhuis J (2011). Couples with unexplained subfertility and unfavorable prognosis: a randomized pilot trial comparing the effectiveness of in vitro fertilization with elective single embryo transfer versus intrauterine insemination with controlled ovarian stimulation. Fertil Steril.

[52] Verhaeghe C, Abnoun S, May-Panloup P, Corroenne R, Legendre G, Descamps P (2020). Conversion of in vitro fertilization cycles to intrauterine inseminations in patients with a poor ovarian response: risk of multiple pregnancies. J Gynecol Obstet Hum Reprod.

